# Evidence for an Association Between Endometriosis and Allergic and Non-allergic Food Hypersensitivity Is Lacking

**DOI:** 10.3389/frph.2021.726598

**Published:** 2021-11-01

**Authors:** Jocelyn O'Malley, Marina Iacovou, Sarah J. Holdsworth-Carson

**Affiliations:** ^1^Department of Obstetrics and Gynaecology, The University of Melbourne and Gynaecology Research Centre, The Royal Women's Hospital, Melbourne, VIC, Australia; ^2^Centre of Innate Immunity and Infectious Diseases, Hudson Institute of Medical Research, Clayton, VIC, Australia; ^3^Department of Molecular and Translational Science, Monash University, Clayton, VIC, Australia

**Keywords:** endometriosis, non-allergic food hypersensitivity, allergic food hypersensitivity, food allergy, food intolerance, comorbidity

## Abstract

Endometriosis effects up to 1 in 9 women, and can be a severe and debilitating disease. It is suggested that there is a link between endometriosis and allergic hypersensitivities, including allergic and non-allergic food hypersensitivity. Best practice for managing endometriosis symptoms is holistic and includes broad multi-disciplinary care. Therefore, improving our understanding of common endometriosis comorbidities, including allergic and non-allergic food hypersensitivity, will assist in improving patient quality of life. This mini-review with systematic approach aims to explore the literature for evidence surrounding an association between endometriosis and allergic and/or non-allergic food hypersensitivity from the last 20 years. Of the 849 publications identified, five fulfilled the inclusion criteria. Only one publication reported a statistically significant increased risk for non-allergic food hypersensitivity in patients with endometriosis (*P* = 0.009), however, the endometriosis group was not uniform in diagnostic criteria and included individuals without laparoscopically visualized disease. No studies elucidated a statistically significant link between allergic food hypersensitivity alone and endometriosis. Therefore, based on a small number of studies with limited research quality, evidence does not support the existence of a link between endometriosis and allergic or non-allergic food hypersensitivity. Sufficiently powered evidence-based research is required, including information which better characterizes the patient's endometriosis symptoms, importantly the gastrointestinal sequalae, as well as specific allergic and non-allergic food hypersensitivities and method of diagnoses. Unequivocally confirming a link between endometriosis and food hypersensitivities is an essential step forward in dispelling the many myths surrounding endometriosis and improving management of disease.

## Introduction

Endometriosis is a common gynecological condition defined by the presence of endometrial-like tissue outside the uterus, which causes scarring and chronic inflammation (1). The pathophysiology of endometriosis remains elusive and is undoubtedly complex, with both genetic and environmental risk factors involved (2). The original theory first opined by Sampson (3) was that of retrograde menstruation causing displacement of endometrial fragments into the peritoneal cavity. Although this explains extrauterine endometrial-like tissue, it does not give the full pathogenesis, as retrograde menstruation occurs in more than 80% of women (4). Recently, a 20 year longitudinal cohort study of over 13,000 Australian women found the prevalence of endometriosis to be as high as 11%, or 1 in 9 (5). Globally, the incidence of endometriosis is immense, and poses a significant public health issue. A greater understanding of the pathophysiology, risk factors, best diagnosis, and treatment pathways are required to better support women with endometriosis.

Symptoms of endometriosis vary considerably in both presentation and intensity, while some individuals are asymptomatic. Endometriosis can present with dysmenorrhoea (cyclical menstrual pain), non-cyclical chronic pelvic pain, deep dyspareunia (painful sexual intercourse) and symptoms related to the bowel or gastrointestinal tract. Bowel symptoms can include constipation, diarrhea, bloating, nausea, vomiting, dyschezia, and blood in the stool (6, 7). To add further burden, women with endometriosis often demonstrate other co-morbid conditions [e.g., cardiovascular disease, autoimmune diseases (including rheumatoid arthritis, Crohn's disease, and ulcerative colitis), irritable bowel syndrome and cancer; (7–10)]. Previous studies suggest that allergic diseases, including atopic conditions such as asthma and rhinitis, and food hypersensitivities are associated with endometriosis (11, 12). For example, one study showed a significantly higher proportion of non-allergic food hypersensitivity, eczema, and hayfever in those diagnosed with endometriosis (11). There is substantial crossover between symptoms associated with food hypersensitivities and common endometriosis-related gastrointestinal symptoms (including abdominal/pelvic pain, dyschezia, bloating, nausea, vomiting, flatulence, and diarrhea) (13, 14). However, evidence supporting the relationship between endometriosis and food hypersensitivity is not well-established. Therefore, based on overlapping symptomology we are yet to fully understand if the disorders truly co-exist or appreciate the potential cause and effect.

Food hypersensitivity can be grouped into two broad categories; allergic and non-allergic food hypersensitivities, which are distinct conditions with different pathophysiologies. Allergic food hypersensitivity occurs when the immune system reacts to allergen(s), which are usually harmless, but the inappropriate immune response leads to antibody production (14, 15). Allergic food hypersensitivity immune reactions can be either IgE mediated, causing an immediate response, or non-IgE mediated, resulting in a delayed response (14). IgE mediated food allergies typically result in angioedema (swelling), wheeze, and urticaria (hives), whereas non-IgE mediated types primarily result in gastrointestinal symptoms (14, 16). Common allergenic foods include eggs, cow's milk, peanuts, tree nuts, soy, wheat, crustacean shellfish, and fish (14, 15). Conversely, non-allergic food hypersensitivity (often referred to as food intolerance) refers to a wide range of conditions that do not involve antibody responses, including reactions to metabolic (enzyme deficiencies), pharmacologic and toxic factors, or idiopathic reactions. Examples of non-allergic food hypersensitivities include lactose intolerance, which is an enzyme deficiency and reactions to chemicals present in foods such as salicylates or monosodium glutamate (14). The symptoms for both allergic and non-allergic food hypersensitivities can present similarly, however, distinction between the two types is vital when considering how they may impact other processes within the body, including comorbid conditions like endometriosis.

Information connecting endometriosis and food hypersensitivity surged in the 1980's, which may have led to the prevailing dogma that allergic and non-allergic food hypersensitivity is more common in women with endometriosis. This quasi-systematic mini-review was designed to explore and consolidate research advances in the literature which associates allergic and non-allergic food hypersensitivities with endometriosis in more recent times (since 2000). We aimed to review the reproducibility, validity and potential limitations of the published research, which will allow us to make recommendations for future studies. Endometriosis patients can experience an extensive list of symptoms and comorbid conditions, and by increasing our understanding of the relationship between endometriosis and food hypersensitivity, we hope to alleviate misinformation and improve disease management which will ultimately enhance the day-to-day lives of those living with endometriosis.

## Methods

### Search Strategy and Selection Criteria

Using a systematic approach, an online literature search of PubMed, Web of Science, and Medline for relevant studies published between 2000 and 2020 was conducted (accessed 2 January 2021). The same keyword search terms and Boolean operators were used for each search engine (allerg*, intolera*, and hypersensitiv* were combined with OR. food, fiber, fat, carbohydrate, sugar, iron, protein, and vegetarian, with OR. endometriosis, period pain, and menstrual pain). Each were combined using AND. Filters for English language and human studies were also applied.

Following removal of duplicates, the titles, and abstracts of each paper were screened for mention of hypersensitivity, allergy or intolerance, and diet control in conjunction with endometriosis. Records were screened by title and abstract and papers fulfilling these selection criteria were read in full and screened more specifically. Papers that did not meet the inclusion criteria consisted of: food hypersensitivity/food allergy/food intolerance not associated with endometriosis; non-food-related hypersensitivity/allergy/intolerance; gastrointestinal symptoms or conditions (for example, irritable bowel syndrome) without mention of food hypersensitivity/allergy/intolerance in association with endometriosis; review papers, systematic reviews, book chapters, and abstract only conference proceedings. Due to the small number of papers meeting these inclusion criteria, none were excluded based on methodology or study design.

## Results

**Figure 1:**
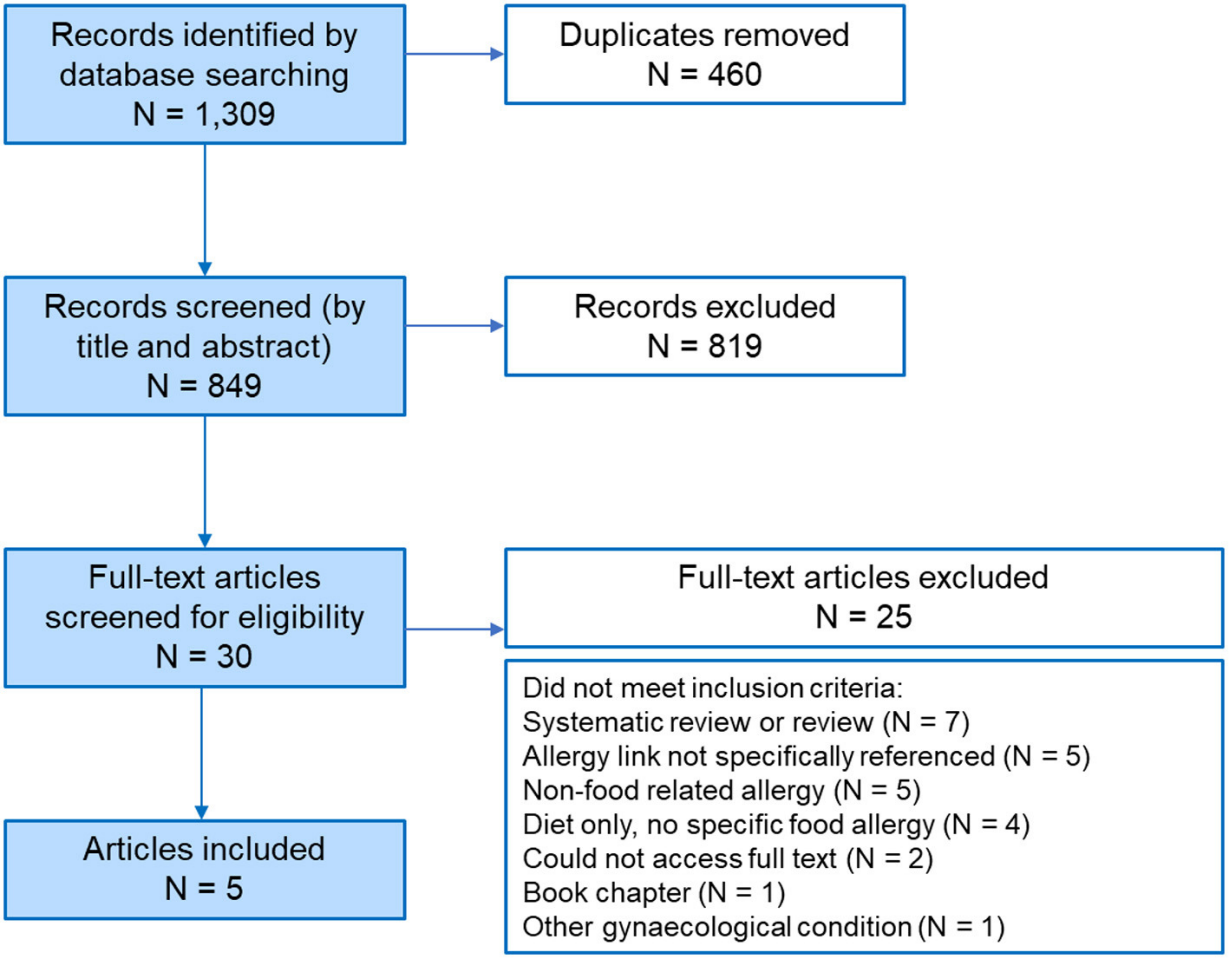
PRISMA diagram. PRISMA flow diagram depicting the search strategy employed to compile this review. In total 1,309 records were reduced to 30 full-text articles, resulting in 5 articles that met the selection criteria outlined in the Methods.

A PRISMA flow chart of the search strategy and selection process employed for this mini-review is shown in Figure 1. After duplicates were removed, 849 publications remained for title and abstract screening. Of these 849, 30 articles remained for full-text screening and only five articles fulfilled the inclusion criteria. The main findings of these articles are presented and summarized in Table 1.

**Table 1:** Summary of articles included in this review.

**Study**	**Study population (N)**	**Endometriosis diagnosis**	**Outcome measure**	**Results**	**Statistical significance**
Sinaii et al. (17)	3,680 (with endometriosis). Prevalence estimates in the general female population (between 1969 and 2001) were selected for comparison to the endometriosis cohort	Laparoscopic	Allergic food hypersensitivity (food merged in an “allergy” group, combined with pollen, dust, trees, paint, grasses, cigarette smoke, perfumes/fragrances, cleaning products, and environmental chemicals)	Combined “allergies” occur in 61% of women with endometriosis vs. 18% of the general population	*P* = < 0.001
Baron et al. (18)	57* (23 with endometriosis, 34 without endometriosis)	Laparoscopic	Non-allergic food hypersensitivity (or dietary intolerance)	Intolerance (non-allergic food hypersensitivity) to 11 various types of foods (accumulative) in women without endometriosis *N* = 45, vs. women with endometriosis *N* = 59 (Nb: individual women could report intolerance to more than one type of food)	No *P*-value published
Baron et al. (19)	54* (22 with endometriosis, 32 without endometriosis)	Laparoscopic	Non-allergic food hypersensitivity (or dietary intolerance)	Intolerance (non-allergic food hypersensitivity) to 11 various types of foods (accumulative) in women without endometriosis *N* = 45, vs. women with endometriosis *N* = 57 (Nb: individual women could report intolerance to more than one type of food)	No *P*-value published
Matalliotakis et al. (20)	689 (501 with endometriosis, 188 without endometriosis)	Laparoscopic	Allergic food hypersensitivity grouped with other allergies (including medications and other drugs; sinus or perennial allergic rhinitis; asthma; other allergies, including dust, pollens, trees, cleaning products, foods and environmental chemicals, and family history of allergic disease)	“Other allergies” including food were found in 14.2% of women with endometriosis vs. 6.9% of those without endometriosis	*P* = 0.010
Schink et al. (21)	208 (156 with endometriosis, 52 without endometriosis)	Clinical or histological	Non-allergic food hypersensitivity (or food intolerance)	Non-allergic food hypersensitivity in 25.6% of endometriosis cases vs. 7.7% of non-endometriosis cases	*P* = 0.009
			Allergic food hypersensitivity	Allergic food hypersensitivity in 15.4% of endometriosis cases vs. 13.5% of non-endometriosis cases	*P* = 0.139

* *Study populations overlap*.

### Endometriosis and Non-allergic Food Hypersensitivity

A recent retrospective case-control study of 208 patients, 156 of whom were diagnosed clinically or histologically with endometriosis, was found to have a significantly higher number of non-allergic food hypersensitivities (reported as food intolerances) in the endometriosis group (*P* = 0.009) compared to the non-endometriosis group (21). Specifically, self-reported intolerances were higher from foods containing sorbitol (*P* = 0.031), histamine (*P* = 0.045), and gluten sensitivity (*P* = 0.025). Two earlier articles by Baron et al., explored the potential link between endometriosis and non-allergic food hypersensitivity (or dietary intolerance) (18, 19). Both papers were based on a prospective comparative study of women who were recruited from the same institution between January 2005 and December 2007; there is apparent replication between papers, although *n* numbers differ slightly between studies without explanation from the authors (Table 1). The studies suggest an increase in non-allergic food hypersensitivity in women with endometriosis in comparison to those without, however this was not of statistical significance. In both studies, the patients were screened for intolerance(s) to various food types which were unusually classified as bread, pizza, pasta, red meat, white meat, chicken, fish, dairy products, milk, yogurt, or vegetables [(18), (Baron, 2012)]. While data was presented for these food types, statistical significance was not calculated.

### Endometriosis and Allergic Food Hypersensitivity

Sinaii et al., in a survey analysis of 3,680 Canadian women with endometriosis looked at the prevalence of allergies; where those reporting one allergy to food, pollens, dust, trees, paint, grasses, cigarette smoke, perfumes/fragrances, cleaning products, or environmental chemicals were combined into one group (17). Overall, statistical significance was found for a higher prevalence of allergies in women with endometriosis relative to the general female USA population [*P* = <0.001; (17)]. Similarly, the paper by Matalliotakis et al., also investigated endometriosis in association with allergies; where allergies were merged as a general broader group of “other” allergies, including those who reported allergic food hypersensitivity (20). This investigation also found a significant increase in the proportion of women with “other” allergies in women with endometriosis compared to controls [*P* = 0.010; (20)]. However, as both papers by Sinnaii et al. (17) and Metalliotakis et al. (20) merge allergic food hypersensitivity with other allergies, specific information separating food allergies from non-food allergies or inherent types of food allergies cannot be deciphered from these works. In contrast, Schink et al., who also reported more women with endometriosis had allergies (*P* = 0.001), did stratify allergies into sub-groups (21). However, allergic food hypersensitivity (food allergy) alone was not found to be statistically significantly higher in women with endometriosis compared those without endometriosis (*P* = 0.139).

## Discussion

### The Origins of Allergic and Non-allergic Food Hypersensitivity in Association With Endometriosis

This mini-review highlights studies from the past 20 years, and struggles to support the association of allergic and/or non-allergic food hypersensitivity in the setting of endometriosis. Two older papers from the mid-1980's were the first to reported a significantly increased risk of allergic diseases in patients with endometriosis than those without (11). Only Lamb and Nichols, found statistical significance specifically to food hypersensitivities, in a case-control study of 86 women (43 with endometriosis) (11). However, no mention was made of how the women with endometriosis were diagnosed (clinically diagnosed, surgical visualization, histopathology, or self-reported). It is concerning that a single, relatively small study, that fails to describe how women were diagnosed with endometriosis, has led to the common conclusion that endometriosis is associated with allergic and non-allergic food hypersensitivities.

### Is There Evidence for a Link Between Allergic Food Hypersensitivity, Non-allergic Food Hypersensitivity, and Endometriosis?

There were major limitations in the collection of dietary information and the classification of allergic and non-allergic food hypersensitivity categories. For instance, two papers exploring non-allergic food hypersensitivity included “white meat,” “chicken,” and “fish” as separate categories, all of which are white meats [(18), (Baron, 2012)]. Non-allergic food hypersensitivity (or food intolerance) was reported to be positively correlated with endometriosis in three out of five papers [(18, 21), (Baron, 2012)], but only one was of statistical significance (21). Two papers exploring a correlation between endometriosis and allergic food hypersensitivity combined food-allergy with non-food related allergy (17, 20) and as such, it is impossible to draw conclusions specifically about food hypersensitivity and endometriosis Finally, all papers, whether retrospective in design or not, collected data detailing endometriosis, dietary intolerance, and/or food allergy using questionnaires/surveys/interviews completed by patients, which could lead to recall bias. It is also reasonable to speculate that prior knowledge of endometriosis diagnosis, as per Sinaii et al., Matalliotakis et al., and Schink et al., could cause participants to have a heightened awareness of their symptomatology including bowel and gastrointestinal issues that maybe associated with both endometriosis and food related hypersensitivities. As such, there is a real lack of definitive association between allergic food hypersensitivity, non-allergic food hypersensitivity and endometriosis in any of these studies, though perhaps due to study design rather than an actual absence of connection.

### Allergic Food Hypersensitivity, Non-allergic Food Hypersensitivity, or Gastrointestinal Symptom of Endometriosis?

Four out of five papers in this mini-review only included women with endometriosis that was laparoscopically or histologically diagnosed [(17, 18, 20), (Baron, 2012)], while one study included patients who were clinically diagnosed based on symptoms alone (21). Symptom-based diagnosis of endometriosis alone cannot be relied upon, as symptoms vary and have considerable cross over with other conditions. As already outlined, endometriosis can present with gastrointestinal symptoms, which are similar to those experienced by those suffering from allergic and non-allergic food hypersensitivities, irritable bowel disease (IBD), Celiac disease, and irritable bowel syndrome (IBS). As of yet, there is no widely accepted clinical criteria for the diagnosis of endometriosis based only on symptoms (22). We note that no studies included in this review investigated potential links between food hypersensitivity with endometriosis stage according to rASRM stage of disease (23). The significant diagnostic challenge for treating clinicians, faced with patients with symptoms that can otherwise be accounted for, is widely acknowledged as a limitation in the endometriosis field. The frustration associated with the lack of non-invasive diagnostic procedures could be alleviated by better understanding the relationships between symptoms and comorbidity risk.

While IBS and other chronic intestinal inflammatory disorders (including IBD and Celiac disease) were outside the inclusion criteria of this mini-review, we recognize the similarity between the symptoms of these conditions, food hypersensitivities, and endometriosis. Acknowledging that Celiac disease is also an autoimmune disease; we accept that several autoimmune diseases have credible associations with endometriosis (24). As reviewed by Turnbull et al., it is not uncommon for patients to be incorrectly diagnosed with IBS or another disorder without further investigation (14), meaning that both endometriosis and/or food hypersensitivities could be misdiagnosed as IBS. Conversely, one must also consider diagnostic bias, whereby those with gastrointestinal symptoms may be more likely to have their endometriosis diagnosed because of increased medical follow-up (or vice-versa in the setting where endometriosis was diagnosed first), thus increasing the co-occurrence of endometriosis and gastrointestinal symptoms falsely. Without clear differentiation in study questionnaires this could easily cause confusion for participants surrounding what is allergic food hypersensitivity, what is non-allergic food hypersensitivity, what is IBS/IBD, and what are symptoms of endometriosis? This is further compounded by the fact no study in the present mini-review required the participants to outline how the diagnosis of food hypersensitivity (allergy or intolerance) was made or whether they had a formal diagnosis of IBS. This raises several questions; do these patients have a food hypersensitivity or IBS, or do they have gastrointestinal manifestations of endometriosis that have been mistakenly defined as food allergy or food intolerance prior to diagnosis of endometriosis, or do they have comorbid conditions?

### Are We Asking About the Right Foods?

Only three of the included papers listed specific foods related to intolerance; sorbitol, histamine, and gluten sensitivity in one (21), and more non-specific (yet inadequately classified) food items such as bread, pizza, white meat, dairy products, pasta, and vegetables in two [(18), (Baron, 2012)]. Neither studies published their questionnaires, therefore it is unknown whether these options were listed by the researcher(s) or whether they were self-reported by participants. Major food allergens include egg, cow's milk, peanut, tree nut, soy, wheat, fish, and shellfish (25). Common non-allergic food intolerances include lactose (a disaccharide found in dairy products), fructose (a monosaccharide found in fruits), and polyols (a sugar alcohol found in some fruits and vegetables) (26). Apart from wheat and dairy products, there is little overlap between common allergic and non-allergic food hypersensitivity triggers. A major limitation of the included articles is the lack of inclusion or investigation of common specific food allergies/intolerances. Until investigations better collect dietary information to answer these fundamental questions, the association between endometriosis and food hypersensitivity will remain unknown.

### Limitations and Future Recommendations

There have been few attempts in the last two decades to clarify the relationship between endometriosis and food allergy and food intolerance, and potential mechanisms of action. We have strived to overcome this by reviewing the more current literature associated with this topic, however, inclusion of literature older than 20 years may have contributed further insight. There are of course other limitations to this mini-review. Although the search was exhaustive (849 papers in total), it did not cover all available databases and journals (for example, only English language and human studies). While one author (JO'M) performed the initial database search, a second author (SH-C) confirmed inclusion of papers when the scope was in doubt. Due to the scarcity of the information obtained following our strict inclusion criteria, all relevant data was included despite identification of some flaws in the methodology of the studies. We highlight that future studies in this area must make a clear distinction between the various types of allergic and non-allergic food hypersensitivity and how participants were diagnosed with food-related hypersensitivity (according to current global clinical standards). It is also important to determine the specific foods within the broad categories of allergic and non-allergic classifications that people with and without endometriosis are allergic or intolerant to using validated assessment instruments. Finally, surgical and histologically confirmed diagnosis and further clinical phenotyping of endometriosis is preferrable. For example, incorporating the rASRM scoring tool for the classification of endometriosis stage, understanding whether their endometriosis is recurrent or not, and importantly, the array of endometriosis-related pain symptoms including gastrointestinal sequelae.

## Conclusion

The present mini-review highlights that the literature is scarce, non-specific, and under-powered to be able to unequivocally conclude any definitive link between endometriosis and allergic and non-allergic food hypersensitivity. Therefore, we conclude that the common rationale that patients with endometriosis experience food allergy and/or intolerance at a greater frequency than women without endometriosis is not supported by evidence-based research. There are however, interesting trends and with improved research methodologies future studies may inform us on how we can better manage endometriosis and related comorbid food hypersensitivities. This information will be crucial to exploring the pathophysiology and mechanisms of action that potentially link food hypersensitivities with endometriosis, if any.

## Author Contributions

JO'M, MI, and SH-C developed the protocol, contributed to results interpretation, and edited the manuscript. JO'M conducted the review, synthesized the data, interpreted results, and drafted the manuscript. All authors approved the final version of the manuscript.

## Funding

SH-C was funded by the National Health and Medical Research Council (NHMRC), 2019 Medical Research Future Fund (MRFF) (APP1199715). MI had support of the Victorian State Government Operational Infrastructure Scheme.

## Conflict of Interest

The authors declare that the research was conducted in the absence of any commercial or financial relationships that could be construed as a potential conflict of interest.

## Publisher's Note

All claims expressed in this article are solely those of the authors and do not necessarily represent those of their affiliated organizations, or those of the publisher, the editors and the reviewers. Any product that may be evaluated in this article, or claim that may be made by its manufacturer, is not guaranteed or endorsed by the publisher.
